# Supplementary material for: Leukocytic Infiltration of Intraductal Carcinoma of the Prostate: An Exploratory Study
Source: Cancers (Basel). 2023 Apr 9;15(8):2217. doi: 10.3390/cancers15082217 (PMC10137226; doi:10.3390/cancers15082217)
Supplement: Supplementary file 1 [file cancers-15-02217-s001.zip › Supplementary Figure S1.pdf]

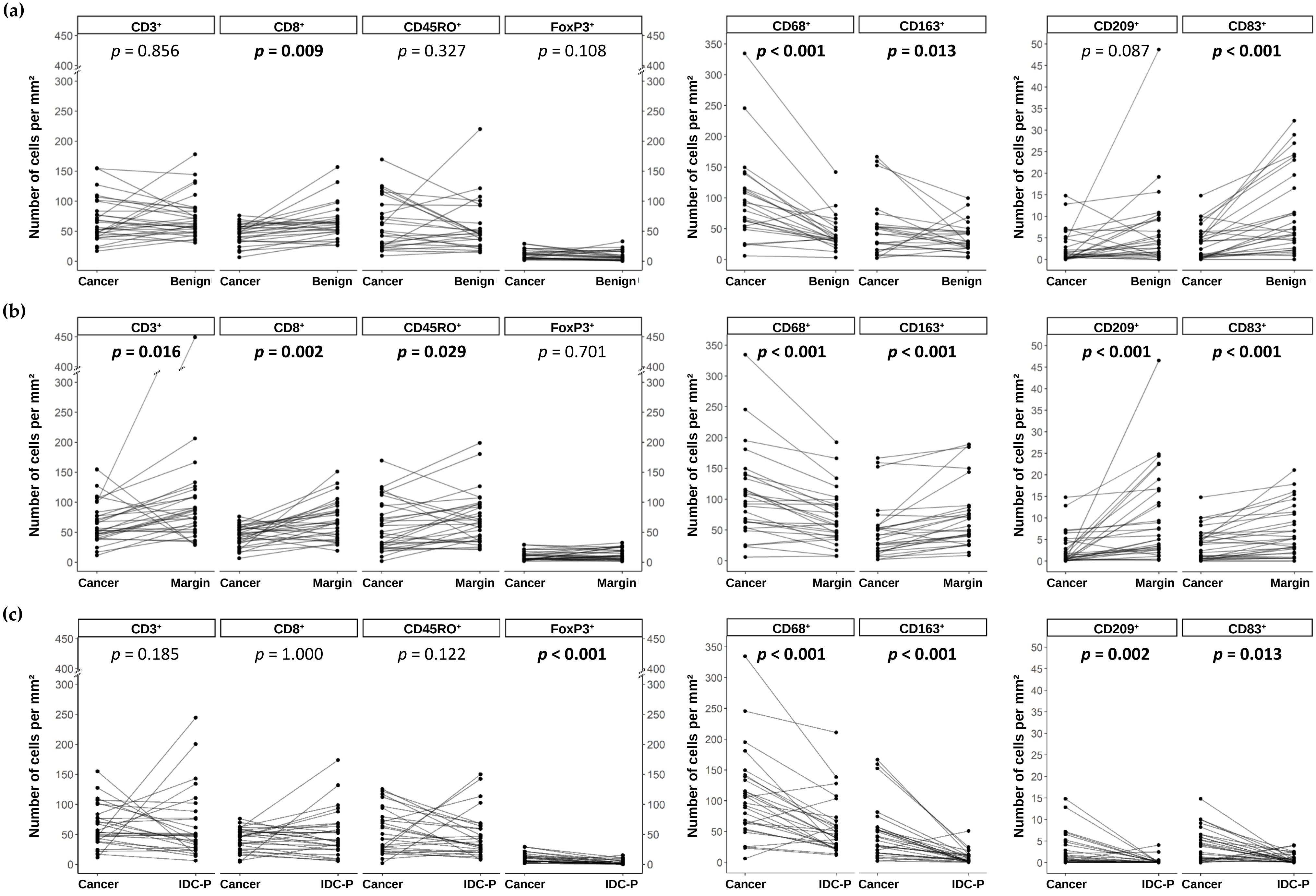

Figure S1. Parallel coordinate plots showing the changes in immune-cell densities between the cancer compartments and benign tissues (**a**), margins (**b**) and IDC-P (**c**) in patients with IDC-P. Paired-samples sign tests were performed. Bold entities indicate statistically significant *p*-values. IDC-P: intraductal carcinoma of the prostate.
